# Green synthesis of selenium nanoparticles using *Cordia myxa* extract and assessment of their cytotoxic and antioxidant properties

**DOI:** 10.22038/ajp.2025.25954

**Published:** 2025

**Authors:** Leili Hosseinpour, Javad Baharara, Majid Darroudi, Zahra Sabouri, aryam Haji Ghasem Kashani, Saeed Zaker Bostanabad

**Affiliations:** 1 *Department of Biology, Mashhad Branch, Islamic Azad University, Mashhad, Iran*; 2 *Nuclear Medicine Research Center, Mashhad University of Medical Sciences, Mashhad, Iran*; 3 *Department of Medical Biotechnology and Nanotechnology, Faculty of Medicine, Mashhad University of Medical Sciences, Mashhad, Iran*; 4 *Department of Cellular and Molecular Biology, School of Biology and Institute of Biological Sciences, Damghan University, Damghan, Iran*; 5 *Department of Biology, Parand Branch, Islamic Azad University, Tehran, Iran*

**Keywords:** Nanoparticle, Cancer, Apoptosis, Biosynthesis, C. myxa plant

## Abstract

**Objective:**

This research aimed to explore the cytotoxic, apoptotic, and antioxidant effects of selenium nanoparticles (Se-NPs) produced by *Cordia myxa* (*C. myxa*) aqueous extract via a green reduction method.

**Materials and Methods:**

To synthesize Se-NPs, Na_2_SeO_3_ and *C. myxa* plant extract were used and their anticancer and antioxidant effects were investigated. After the synthesis of Se-NPs, their physicochemical properties were investigated by various techniques such as UV-Vis, XRD, TEM, FESEM/PSA, EDX, FT-IR, and DLS/Zeta. Next, the lethality of Se-NPs on cancerous Huh-7 and normal L929 cells was investigated. Then, using Annexin V/PI and DAPI kits, the number of apoptotic cells in terms of the obtained percentage and the amount of ROS production was determined by flow cytometry. The influence of Se-NPs on the expression of genes associated with apoptosis has been examined.

**Results:**

The XRD pattern and FESEM/TEM images confirmed the successful production of Se-NPs with an amorphous nature and size average of 11.9 nm. Flow cytometry analyses revealed a significant increase in ROS levels after 24 h of treatment with Se-NPs, demonstrating a concentration-dependent effect.

**Conclusion:**

The findings indicate that Se-NPs exhibit anticancer activity against Huh-7 cells, as evidenced by the upregulation of *Bax, p53, and Caspase3 gene* expression. Therefore, it can be asserted that Se-NPs have the potential to eliminate cancer cells, while simultaneously providing a protective effect on normal cells.

## Introduction

Cancer is a disease that kills a large number of people every year. Many cancer treatment methods, including chemotherapy drugs, in addition to being expensive, cause severe damage to healthy cells in the body and have a wide range of side effects. That is why the priority in conducting this research was to use an effective, inexpensive, tolerable, and low-risk method (Garcia-Oliveira et al. 2021). Cancer is the result of abnormal cell development which is associated with altered cellular physiology; these modifications involve angiogenesis capability, growth signals of autonomy, no restriction in cell division, tissue invasion, and apoptosis resilience. Apoptosis is the planned elimination of cells. Any disruption in the process of apoptosis results in disease (Carneiro and El-Deiry 2020).

Nanoparticles have emerged as a promising method for targeted drug delivery, enabling direct interaction with specific cells within tissues. Two critical properties influencing the choice of surface coating for nanoparticles are colloidal stability and cytotoxicity. In the case of metal nanoparticles, using natural substances is advisable to maintain a stable homogeneous solution, ensuring both regenerative properties and a robust metal nanoparticle coating. This approach aims to mitigate potential risks to human health and the environment. Over the past decade, selenium nanoparticles (Se-NPs) synthesis has garnered significant interest due to their potent antioxidant capabilities. Their diminutive size facilitates interactions with cells and tissues at the molecular level. Se-NPs exhibit several advantageous characteristics for clinical applications including reduction of drug resistance, decreased drug dosage requirements, tumor diagnosis and treatment, bio-compatibility, and bio-degradability. These properties position Se-NPs as valuable tools in cell therapy, tissue repair, and drug delivery systems. The potential of green synthesis, facilitated by the synergistic effects of organic and biodegradable agents derived from plants that possess reducing and scavenging properties in conjunction with specific selenium-like metals, represents one of the most fascinating and significant areas of exploration within the field of nanotechnology (Amiri et al. 2021; Hasanin et al. 2021; Varlamova et al. 2021). So far, plants such as *Rosmarinus officinalis *(Adibian et al. 2022a), *Urtica dioica *(Hashem and Salem 2022), *Ceropegia bulbosa *(Cittrarasu et al. 2021), *Abelmoschus esculentus *(Ghaderi et al. 2022), and many other plants have been used for the biological production of Se-NPs.

This study examined the cytotoxic impact of Se-NPs synthesized using *Cordia myxa* extract on the Huh-7 cells, along with their apoptotic and antioxidant properties. Previously, many nanoparticles such as zinc oxide nanoparticles (Nagaraja and Hwan 2023), silver nanoparticles (Akbarnejad-Samani et al. 2020; Samari et al. 2019), and palladium nanoparticles (Nagaraja et al. 2023) have been produced using *C. myxa *extract. Research has yet to be undertaken regarding the cytotoxicity and antioxidant properties of Se-NPs derived from the plant extract *C. myxa*. The absence of studies on the cytotoxicity and antioxidant effects of Se-NPs synthesized from plant extracts, highlights the innovative nature of this research. So far, studies have been conducted on the anti-cancer effect of green-synthesized metal nanoparticles. The cytotoxicity and antioxidant activity of Se-NPs in *C. myxa* extract on Huh-7 and L929 cell lines have not been investigated (Khojasteh-Taheri et al. 2023; Sabouri et al. 2022a; Sabouri et al. 2022b). *C. myxa *is a tree of the Boraginaceae family with a wide range of biological properties including anti-bacterial, anti-fungal, anti-inflammatory, etc. *C. myxa *extract has not been used for the synthesis of Se-NPs, according to the literature review. The plant* C. myxa* is a member of the order Boraginales, family Boraginaceae, and genus* Cordia*. 

The present study was carried out using the fruits of this plant to synthesize Se-NPs. We hypothesized that the bioactive compounds present in the fruits of plants, especially in *C. myxa* extract, would have the advantages in reduction and stabilization of metal NPs, such as Se-NPs. The strong pharmacological activities of the *C. myxa* extract may be due to the high concentration of polyphenols including flavonoids which have been shown to have strong anti-tumor and antioxidant capacities.

## Materials and Methods

### Materials

MTT Powder, RPMI 1640 (Gibco, USA), penicillin/streptomycin (Sigma, USA), trypsin-EDTA (Neogene, Iran), Na_2_SeO_3_, Fetal bovine serum (FBS), and DMSO (Dimethyl sulfoxide) were acquired from Merck and Sigma. Also, dried fruits of the *C. myxa* plant were obtained from grocery shops in Mashhad, Iran.

### Preparation of C. myxa plant extract

One gram of the dry fruit of *C. myxa* plant was combined with 100 mL of water as a solvent and stirred at 55°C for 2 hr. The resulting light brown liquid was filtered through a filter paper. Then, the freshly filtered extract was used for nanoparticle synthesis (Hosseinpour et al. 2022).

### Synthesis of Se-NPs

**Figure 1:**
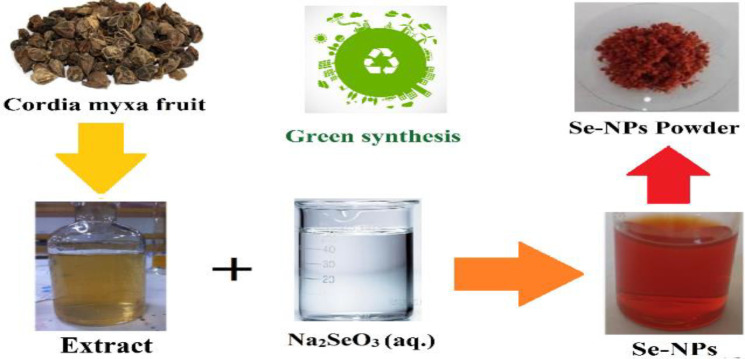
Synthesis of Se-NPs

Nanoparticle synthesis started with 0.263 g of Na_2_SeO_3_ dissolved in 100 mL of water using a stirrer. In the next stage, 20 mL of the extract obtained in the previous step was added gradually and each time 20 μL was added to the prepared salt bed. This solution was stirred for 1 hr (at a temperature of 75°C), after that, the stirring was continued for 48 hr (at a temperature of 26°C). When finished working, an orange-colored colloidal solution was obtained. In this process, the color change from light brown to deep orange indicates the synthesis of Se-NPs. The obtained orange solution was dried by a freeze-dryer (freeze dryer F.D-V-450) at -80°C for 48 hr. (Hosseinpour et al. 2022). A schematic representation of the biosynthesis process of Se-NPs is shown in Figure 1.

### Characterization of nanoparticles

To determine the features of the synthesized nanoparticles, there are a set of analytical strategies including XRD (X-Ray diffraction), UV–Vis (Ultraviolet–Visible),

EDX (energy dispersive X-ray), FTIR (Fourier Transform Infrared Spectroscopy), DLS (Dynamic Light Scattering)/Zeta potential, TEM (Transmission electron microscopy)/FESEM (Field emission scanning electron microscopy), and PSA (Particle size analysis). These techniques can facilitate selective detection of stable Se-NP size and framework. Next, surveying the nonmaterial’s morphology and size was done by FESEM (Hitachi, S4800) and EDX analysis. Furthermore, the stability/size of nanomaterials was examined in terms of the DLS/Zeta potential. In addition, FTIR analysis (Shimadzu 8400, Japan) was utilized to identify the functional groups present in the Se-NPs (Adibian et al. 2022a). Also, the size of nanoparticles was estimated from the images of the TEM and FESEM, using the ImageJ software.

### Cell culture

We obtained the Huh-7 and L929 cell lines from the Pasteur Institute in Tehran, Iran. The cells were cultured in a complete RPMI 1640 medium which included 10% FBS and 1% penicillin-streptomycin. This culture process was conducted in specialized cell culture flasks and incubated in a CO_2_ environment maintained at 5%, with a relative humidity of 95% at a temperature of 37°C (Hosseinpour et al. 2022).

### MTT assay

The cytotoxic effects of the synthesized Se-NPs were assessed on Huh-7 liver cell lines utilizing the MTT assay. Initially, the cell line was cultured in 96-well plates using RPMI medium supplemented with 10% FBS, along with penicillin (100 units/mL) and streptomycin (100 μg/mL) as antibiotics, and then incubated. After 24 hr incubation period to ensure cell adherence to the plates, the cells were treated with varying concentrations of Se-NPs (15.5 to 500 μg/mL). Following an additional 24 hr, 20 μL of MTT solution at a concentration of 5 mg/mL was introduced to each well, and the incubation continued at 37°C for 4 hr. Subsequently, 100 μL of DMSO was added to each well and allowed to incubate for 15 min at room temperature. The final measurement of absorbance was conducted using an ELISA reader set to a wavelength of 570 nm. Each experiment was performed in quadruplicate to enhance the reliability and accuracy of the results. The final analysis of cell viability and the determination of IC_50_ values were carried out using SPSS^®^ and Prism^®^ software (Sabouri et al. 2021a).

### DAPI nuclear staining

DAPI (4′,6-diamidino-2-phenylindole) is a fluorescent dye, exhibits a strong affinity for adenine-thymine-rich regions within DNA, facilitating the analysis of nuclear morphology through fluorescence microscopy. Initially, 35×10^4^ Huh-7 cells were cultured in each well of a 6-well plate to assess the effects of varying concentrations of Se-NPs (20, 60, and 100 µg/mL) over a 24 hr period. Following this incubation, 1.0 mL of DAPI solution was introduced to each well and allowed to incubate for 15 min. Subsequently, the DAPI solution was removed, and 1 mL of methanol was added to each well to promote cell sedimentation (Pasha et al. 2022). All working steps were carried out in the darkness, and the morphology of the cell nucleus was studied under a fluorescence microscope (Biomed, Korea).

### Measurement of oxygen free radical production

The evaluation of the effects of Se-NPs on the induction of oxidative stress and reactive oxygen species (ROS) production in Huh-7 cancer cells necessitated the use of the DCFDA/H2DCFDA kit, a cellular ROS detection assay provided by Abcam. This involved the cultivation of 20,000 Huh-7 cells in each well of a 96-well plate, followed by treatment with Se-NPs at concentrations of 20, 60, and 100 µg/mL for a duration of 24 hr. Post-treatment, the cells were stained with DCFDA at a concentration of 25 µM under dark conditions at 37°C for 30 min, as outlined by Afshari et al. (Afshari et al. 2019). 

Subsequently, fluorescence measurements were obtained using a PERKINELMER fluorometer, with excitation and emission wavelengths set at 485 and 535 nm, respectively.

### Annexin 5-propidium iodide assay for the detection of apoptosis

The translocation of phosphatidylserine from the inner to the outer leaflet of the cell membrane serves as a significant biomarker for the apoptotic process. To assess this phenomenon, an Annexin V propidium iodide kit (Abcam) was employed. Upon the externalization of phosphatidylserine on the cell surface, it can be identified using a fluorescent dye that specifically binds to Annexin V. Initially, 35×10^4^ cells (Huh-7) were cultured in each well of a 6-well plate, followed by treatment with varying concentrations of Se-NPs at 20, 60, and 100 μg/mL for a duration of 24 hr. After this incubation period, the cells were trypsinized and subjected to centrifugation (Bruker Avance 3 300 MHz) at 400 g for 5 min. Subsequently, 200 μL of Annexin-Binding buffer (1x) was added to the cell pellet, and the mixture was centrifuged once more. In the following step, 50 μL of Annexin V-FITC/PI solution was introduced to the cell sediment and allowed to incubate for 10 min at room temperature in the absence of light. Finally, 150 μL of Annexin-Binding buffer (1x) was added to the resulting suspension which was then analyzed using flow cytometry (BD, USA) (Amiri et al. 2021).

### Gene expression analysis

A real-time PCR assay was employed to assess the expression levels of the *Bax, p53, Caspase 3, and Caspase 9* genes. Following the extraction of RNA using an RNA extraction kit (Pars Tous, Iran), the RNA concentration necessary for the synthesis of complementary DNA (cDNA) was quantified with a Nanodrop device at wavelengths of 260 and 280 nm. Subsequently, cDNA synthesis was conducted in accordance with the protocol provided by the cDNA kit (Pars Tous, Iran). Finally, the real-time PCR analysis was performed utilizing the Cybergreen kit (SYBR Green Master Mix, Pars Tous, Iran) (Amiri et al. 2021). In this study, the primers targeting the *Bax, p53, Caspase 3,* and *Caspase 9* genes were designated as the target genes, while the GAPDH gene served as the internal control (Table 1)

### Statistical analysis

A one-way analysis of variance (ANOVA) was conducted alongside Tukey's multiple comparisons test for the statistical evaluation. A significance level of p≤0.05 was established for the analysis. All statistical computations were carried out using GraphPad Prism version 8.0.2.

**Table 1:** Primers sequence.

Primers (5ʹ → 3ʹ)	Gene name	Gene symbol
**Forward: ** **CAAACTGGTGCTCAAGGCCC** **Reverse:** **GAAGTCCAATGTCCAGCCCA**	*Bcl-2-associated X protein*	*Bax*
**Forward: ** **AACAGCTTTGAGGTGCGTGT** **Reverse: ** **GTTGGGCAGTGCTCGCTT**	*Tumor suppressor protein*	*P53*
**Forward: ** **TGGAATTGATGCGTGATGTTTCT** **Reverse: ** **ACTTCTACAACGATCCCCTCTG**	*Cysteine-aspartic acid protease 3*	*Caspase3*
**Forward: ** **TCCTACTCTACTTTCCCAGGTTT** **Reverse:** **AAAGCAACCAGGCATCTGTT**	*Cysteine-aspartic acid protease 9*	*Caspase9*
**Forward: ** **TGGAAGGACTCATGACCACAGT** **Reverse:** **TTCCCGTTCAGCTCAGGGAT**	*Glyceraldehyde-3-phosphate dehydrogenase*	*GAPDH*

## Results

### XRD

**Figure 2:**
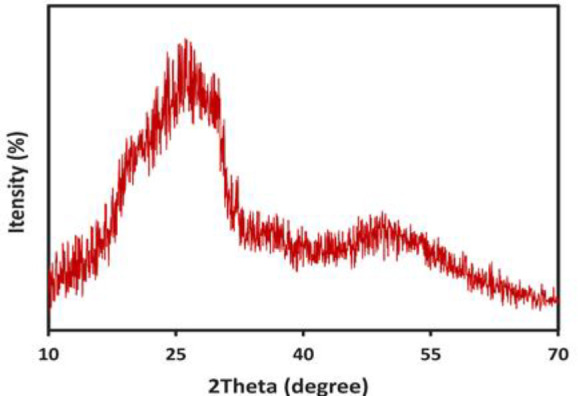
The XRD pattern of Se-NPs.

The characterization of the Se-NPs in terms of their structure, purity, and crystallite size was conducted through XRD analysis, as illustrated in Figure 2. A comparative analysis of the XRD pattern of the synthesized Se-NPs with the standard JCPDS (Card No # 06-362) (Alagesan and Venugopal 2019; Cittrarasu et al. 2021) revealed that the synthesized Se-NPs are entirely pure and exhibit an amorphous structure. The absence of distinct, narrow high-intensity peaks in the XRD pattern further corroborated their amorphous characteristics. These findings were aligned with the observations reported by Alagesan *et al*. (Alagesan and Venugopal 2019).

### UV-Vis/Bandgap

**Figure 3:**
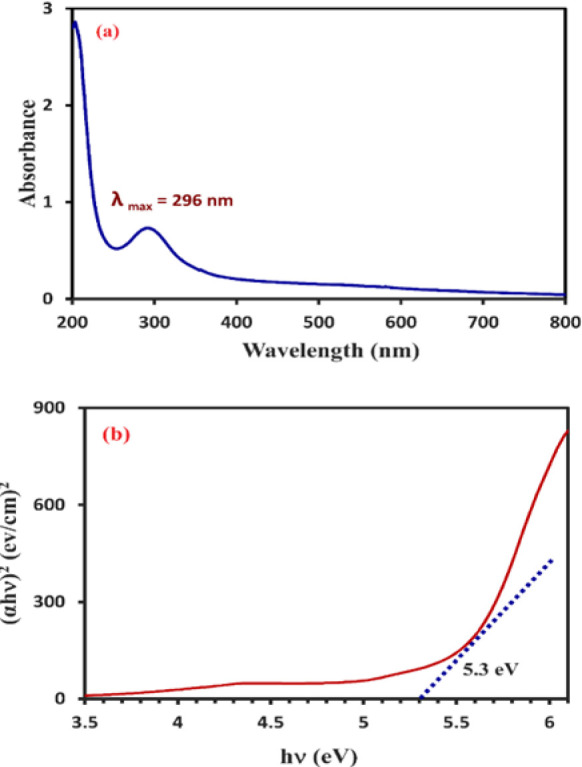
UV-Vis (a) and bandgap (b) of Se-NPs

The UV-Vis spectrum was obtained across the full range of 200-800 nm to verify the synthesis of Se-NPs. The presence of Se-NPs in the UV-Visible spectrum at 296 nm (Figure 3a), as indicated by the data derived from surface plasmon resonance (SPR), substantiates their formation. Consistent with contemporary studies, the closeness of the conduction and valence bands facilitates the movement of electrons, which may contribute to an enhancement in the SPR absorption band (Cittrarasu et al. 2021; Sabouri et al. 2021b; Velayati et al. 2021b). In alignment with the UV-Vis findings, the bandgap energy (Eg) of the nanoparticles, calculated using Equation 1, was approximately 5.3 eV. The plot of (ahν) n against hν is presented in Figure 3b. These results corroborate the observations made by Velayati et al. (Velayati et al. 2021a; Velayati et al. 2021b; Velayati et al. 2022). 

$\left(\mathrm{\alpha h\nu}\right)={A\left(\mathrm{h\nu}-E_{g}\right)}^{n}$ (1)

In this context, ν represents the light frequency, while α denotes the light absorption coefficient. The constants A and E(g) remain fixed, with the values of n and the band gap being determined by the specific type of transition occurring in the Se-NPs.

### DLS analysis and Zeta potential

**Figure 4:**
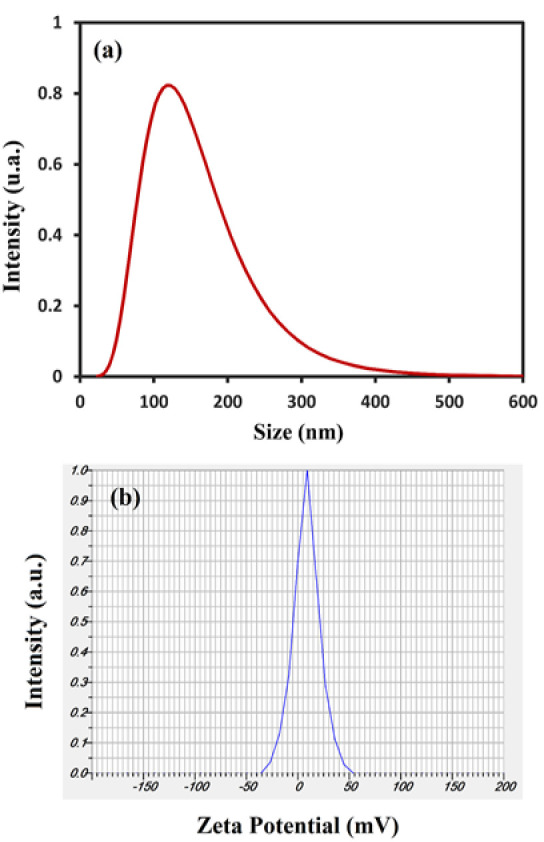
DLS analysis (a) and Zeta potential (b) of Se-NPs

Dynamic Light Scattering (DLS) enables the computation of p estimates for dispersed Se-NPs throughout the solution (Figure 4 a and b). According to DLS analysis, the nanoparticles exhibit an average size of approximately 117 nm, accompanied by a polydispersity index (PDI) of 0.197. It is well established that zeta potential refers to the actual electric charge present in the vicinity of the nanoparticle's surface. Our results align with the findings reported by Adibian *et al*. and Ghaderi* et al*. (Adibian et al. 2022b; Ghaderi et al. 2021). 

### TEM image

**Figure 5:**
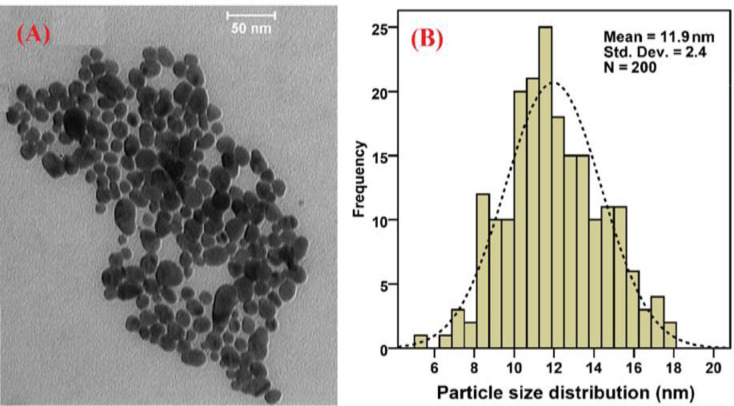
Results of TEM (a) and PSA (b) of Se-NPs

The morphology and size of the synthesized NPs were investigated by TEM/PSA imaging. The results obtained from this imaging showed a round framework of NPs (Figure 5A) that were finely dispersed when synthesized, while the PSA histogram (Figure 5B) demonstrated that the size of NPs is around 11.9 nm. 

### FESEM/EDX/PSA

**Figure 6:**
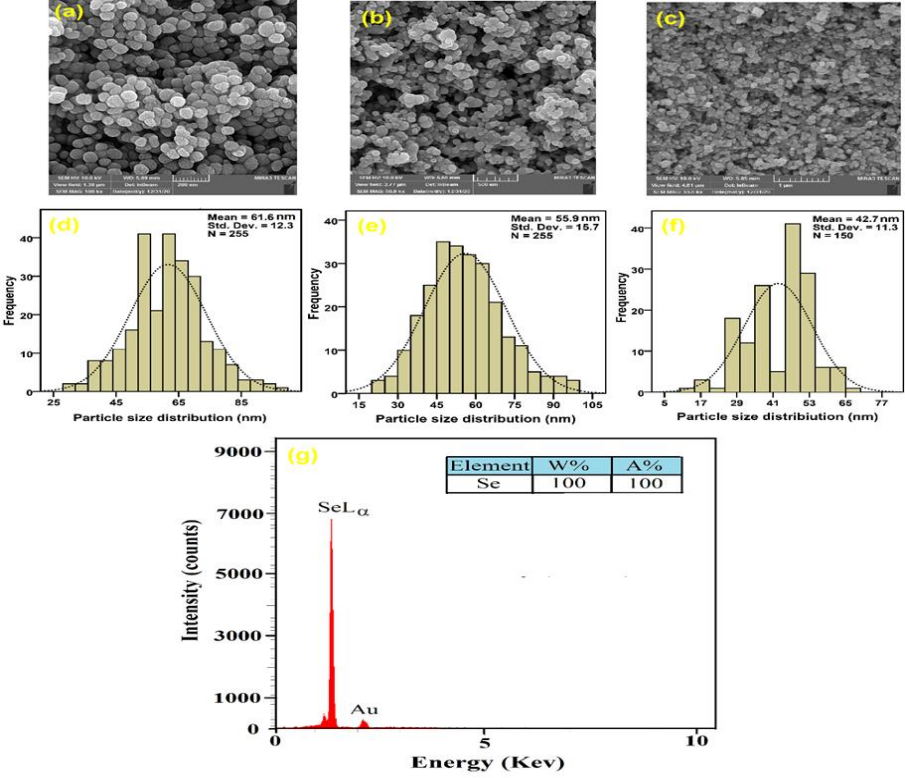
The FESEM image (a-c), particle size distribution (d-f), and EDX analysis (g) of Se-NPs

The displayed pictures of FESEM (Figure 6 a-c) affirmed the circular shapes of our product. The presence of this element was confirmed by the selenium peak in the EDX test (Figure 6g). The EDX examination showed gold peaks as a result of the coated gold within the network (Adibian et al. 2022b). The results of this work are similar to the reports of Ghaderi et al. (Ghaderi et al. 2021). The size of Se-NPs was about 40-60 nm (Figure 6 d-f).

### FTIR

**Figure 7:**
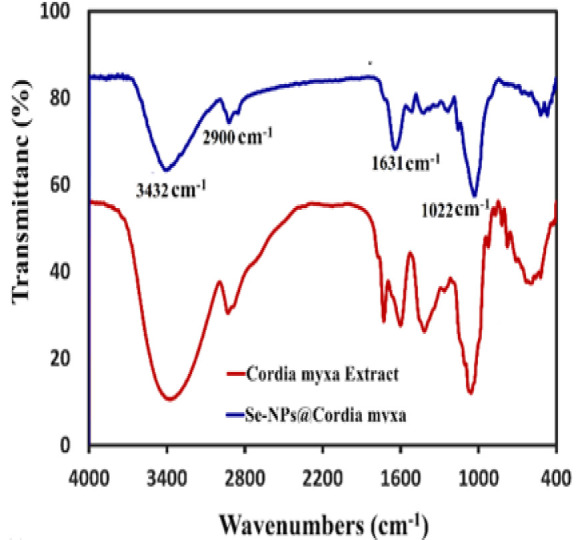
FTIR spectrum of C. myxa extract and Se-NPs

To investigate the presence of a stabilizer (*C. myxa* extract) in the Se-NPs solution, FTIR analysis was conducted over a range of 400-4000 cm^-1^, with the results illustrated in Figure 7. The FTIR spectrum of the *C. myxa* extract reveals a prominent peak at 3375 cm^-1^, indicative of the stretching vibration of the hydroxyl (OH) group, while a peak at 2920 cm^-1^ corresponds to the stretching vibration of the methylene (CH) group. Additionally, the absorption bands for the carboxylate (COO-) and ether (C-O-C) groups are observed between 1005 and 1730 cm^-1^. In the FTIR spectrum of the Se-NPs, a notable shift of the OH group absorption band from 3375 to 3400 cm^-1^ is evident, accompanied by a reduction in peak intensity, suggesting that the free hydroxyl groups present in the *C. myxa* extract are interacting with the Se-NPs. Furthermore, the intensity of the C-H vibration peak at 2920 cm^-1^ in the Se-NPs is slightly diminished compared to that in the *C. myxa* extract. The bands observed at 1003 and 1640 cm^-1^ are attributed to the stretching vibrations of the COO- and C-O-C groups, with a similar decrease in peak intensity for the Se-NPs relative to the extract. Consequently, the FTIR spectrum of the Se-NPs closely resembles that of the *C. myxa* extract, providing evidence of the interaction between the compounds in the *C. myxa* extract and the Se-NPs, thereby indicating that the stabilizer is integrated within the Se-NPs and suggesting that *C. myxa* may play a protective role against the accumulation of Se-NPs (Sabouri et al. 2021a).

### In vitro study of Se-NPs

**Figure 8:**
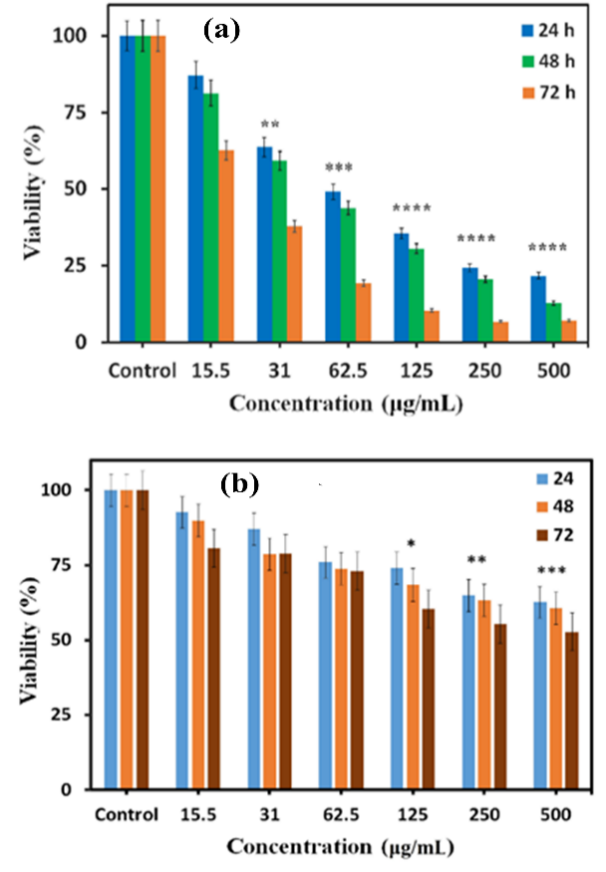
The viability of Se-NPs towards Huh-7 (a) and L929 (b) cells was compared using one-way ANOVA (*p<0.05, **p<0.01, ***p<0.001, and ****p<0.0001 as compared with control group).

In this study, the survival rates of Huh-7 cancer cells and normal L929 cells treated with Se-NPs, were evaluated. The findings demonstrate a concentration- and time-dependent inhibitory effect of Se-NPs on the proliferation of liver cancer cells. Specifically, the IC_50_ values for Huh-7 cells were approximately 62.5 µg/mL after 24 and 48 hr, and around 31 µg/mL after 72 hr (see Figure 8a). These calculations suggest that the level of toxicity is directly related to the concentration of Se-NPs, with toxicity decreasing as the concentration is reduced. Furthermore, the assessment of Se-NPs' cytotoxicity on the normal L929 cell line revealed that these nanoparticles exhibit moderate toxicity at elevated concentrations (500 and 250 µg/mL) and prolonged exposure (72 hr), while demonstrating minimal toxicity at lower concentrations (refer to Figure 8b). Previous studies have explored the toxic effects of Se-NPs on various cancer cell lines (Hasanin et al. 2021; Varlamova et al. 2021). However, the specific cytotoxic effects of our formulation on the Huh-7 liver cancer cell line remain unexplored. 

### DAPI staining results

**Figure 9:**
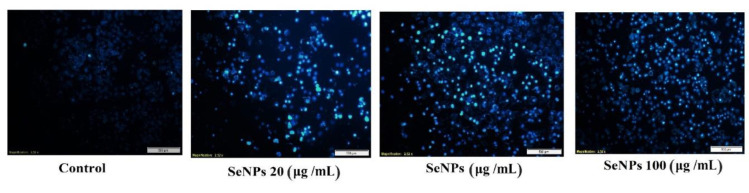
Qualitative effect of apoptosis induction by Se-NPs using DAPI staining method on Huh-7 cells

The DAPI staining results indicated that varying concentrations of our synthesized product, derived from *C. myxa* extract (20, 60, and 100 µg/mL), led to nuclear fragmentation, a hallmark of apoptosis in the cells. As illustrated in Figure 9, the extent of these cellular alterations intensified with higher concentrations of Se-NPs, with the most pronounced fluorescence observed at 100 µg/mL.

### Se-NPs induce apoptosis on Huh-7 cell line

**Figure 10:**
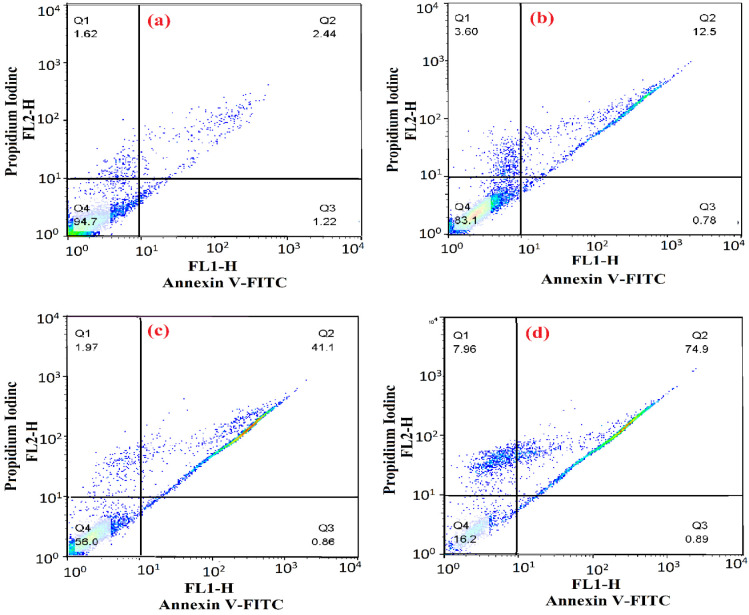
Apoptotic functional of Se-NPs on Huh-7 cell line. a: flow cytometric result of Huh-7 cancer cell as the control without the presence of Se-NPs. b-d: flow cytometric results of the effect of Se-NPs synthesized with C. myxa extract (20, 60, and 100 μg/mL) after 24 hr. (Q1: necrotic cells, Q2: late apoptotic, Q3: early apoptotic, and Q4: viable cells).

**Figure 11:**
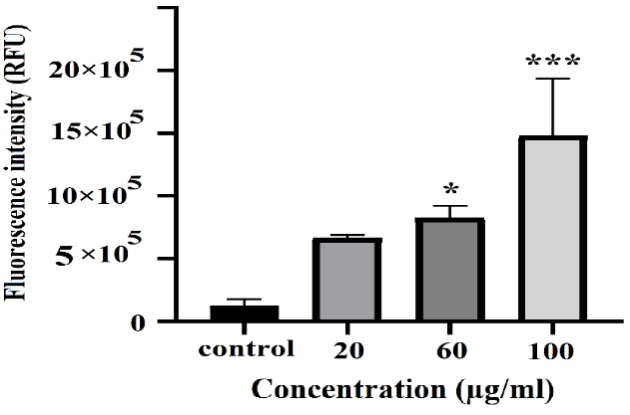
The percentage of oxygen free radicals’ production after Se-NPs treatment on Huh-7 cells after 24 hr of incubation was compared by one-way ANOVA analysis (p*0.01 and p<0.0001 as compared to control group).

At this juncture, the process of apoptosis was examined utilizing an Annexin-V and PI double staining kit on the Huh-7 cell line (Figure 10). In the present study, we examined the impact of Se-NPs on ROS production in Huh-7 cancer cells. Flow cytometry analyses revealed a significant increase in ROS levels after 24 h of treatment with Se-NPs, demonstrating a concentration-dependent effect (Figure 11). 

### Gene expression analysis

**Figure 12:**
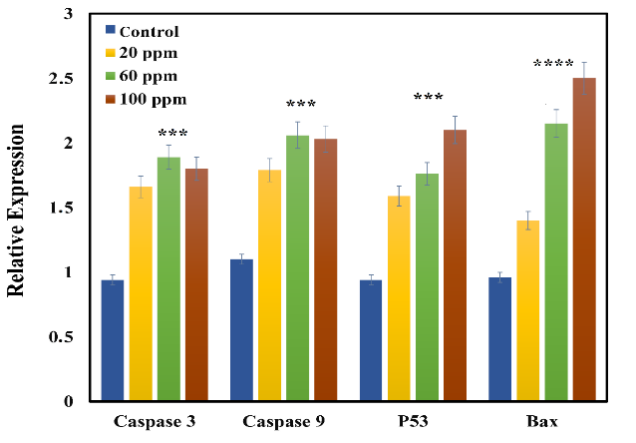
The expression percentage of Bax, p53, Caspase3 and Caspase9 genes after Se-NPs treatment on Huh-7 cells was compared by one-way ANOVA analysis (*p<0.05, **p<0.01, ***p<0.001, and ****p<0.0001 as compared to control group).

The results obtained from Real-Time PCR indicate that Se-NPs have a substantial impact on the expression of apoptotic genes. Specifically, the treatment of Huh-7 cells with Se-NPs seems to increase the expression levels of *Bax, p53, caspase3, and caspase9* when compared to the GAPDH reference gene (Figure 12).

## Discussion

The findings of this study provide evidence that Se-NPs may induce cancer cell death, as demonstrated by flow cytometry data, the observed anti-cancer effects against Huh-7 cells, and the upregulation of pro-apoptotic genes. Conversely, these nanoparticles exhibit antioxidant properties in normal cells, thereby offering a protective effect. The remarkable antioxidant capacity of plant-derived Se-NPs is linked to their elevated selenium content, which is crucial for enhancing the activity of selenium-dependent enzymes such as glutathione peroxidase and thioredoxin reductase, thus safeguarding cells and tissues from oxidative stress. Consequently, the Se-NPs synthesized in this research present a promising strategy for the treatment of liver cancer.

A principal mechanism through which nanoparticles, such as Se-NPs, exert cytotoxic effects and induce cell death in cancer cells is their capacity to generate ROS and provoke oxidative stress resulting from the accumulation of nanoparticles within these cells. Evidence suggests that cancer cells generate higher levels of ROS compared to normal cells, rendering them more susceptible to oxidative stress. Prior studies conducted in both *in vitro* and i*n vivo* settings have demonstrated that Se-NPs can effectively eliminate cancer cells and inhibit tumor proliferation, primarily due to a marked increase in ROS production (Wu et al. 2019). The anticancer properties of Se-NPs warrant further exploration and analysis; however, existing research, including this study, indicates that Se-NPs can stimulate the proliferation of healthy cells while simultaneously inhibiting the growth of malignant cells (He et al. 2025; Nagime et al. 2025). Due to their diminutive size, these nanoparticles can traverse the plasma membrane through receptor-mediated endocytosis. Cancer cells typically exhibit an acidic pH and a redox imbalance, conditions that facilitate the generation of free radicals by Se-NPs. This increase in free radicals can compromise the integrity of the mitochondrial membrane, leading to the leakage of mitochondrial proteins and the induction of endoplasmic reticulum stress. The disruption of mitochondrial membranes and the subsequent release of proteins activate *caspases*, culminating in a series of detrimental cellular events that result in DNA fragmentation, cell cycle arrest, and ultimately, apoptosis (Lobb 2011; Naseer et al. 2022). It is also hypothesized that the activation of *p53*, along with increased *Bax* levels, may facilitate the release of apoptosis-inducing factors from the mitochondria, such as cytochrome C, ultimately leading to the activation of the *caspase* cascade. The findings of the current study indicate that the observed increase in the expression of these genes correlates with the presence of Se-NPs, consistent with earlier research (Figure 12) (Almarzoug et al. 2020; Baharara et al. 2016; Katifelis et al. 2018).

Previous research has established that the *p53* protein is crucial in mediating cellular responses to DNA damage and apoptosis, primarily through the enhancement of ROS and oxidative stress. Typically, *p53* remains inactive within cells, maintaining low concentrations. However, under stress conditions, an upregulation of *p53* expression can halt the cell cycle, providing an opportunity for DNA repair or triggering apoptosis. Furthermore, *p53* can activate the mitochondrial apoptotic pathway by downregulating the anti-apoptotic protein *Bcl-2* while promoting the expression of the pro-apoptotic protein *Bax*. Previous studies have indicated that Se-NPs exhibit selective anti-cancer properties against malignant cells while demonstrating minimal toxicity to healthy cells (Ullah et al. 2022). In the context of drug delivery and oncological treatment, conventional anti-cancer agents cannot often differentiate between normal and cancerous cells, necessitating the administration of high doses to effectively target tumor sites. This elevated dosage can result in significant adverse effects. Research has shown that nanostructures with hydrophilic surfaces, particularly those under 100 nm in size, possess enhanced evasion capabilities from the molecular phagocytic system, which includes endoplasmic reticulum organelles that can rapidly engulf hydrophobic nanoparticles. The findings of this investigation reveal that Se-NPs are hydrophilic and approximately 11.9 nm in diameter, allowing them to circulate in the bloodstream for prolonged periods. Recent assessments indicate that low concentrations of these nanomaterials exhibit considerable biocompatibility, whereas higher doses pose a substantial risk to normal or healthy cells (Lin et al. 2021).

Our findings suggest that the apoptosis observed in liver cancer cells treated with Se-NPs is likely attributable to elevated ROS production and subsequent oxidative damage, which may activate apoptotic pathways. Moreover, Se-NPs have the potential to induce various forms of cell death, including autophagy and necrosis, in cancer cells. Consistent with our findings, an increase in Se-NP concentration in the Huh-7 cell line corresponded with a rise in secondary apoptosis, characterized by the translocation of phosphatidylserine from the inner to the outer leaflet of the plasma membrane, indicative of cellular disruption. Further exploration of the molecular mechanisms involved indicates that apoptosis in this context is mediated by the activation of *caspase-3,* as illustrated in Figures 10 and 11. These observations align with findings documented in earlier research (Baharara et al. 2016; Wu et al. 2019).

## Abbreviations

XRD (X-Ray diffraction)

UV–Vis (Ultraviolet–Visible)

EDX (energy dispersive X-ray)

FTIR(Fourier Transform Infrared Spectroscopy)

DLS (Dynamic Light Scattering)/Zeta Potential

TEM (Transmission electron microscopy)

FESEM (Field emission scanning electron microscopy)

PSA (Particle size analysis)

PDI (Polydispersity index) 

DMSO (Dimethyl sulfoxide) 

FBS (Fetal bovine serum)

DAPI (4′,6-diamidino-2-phenylindole) 

Se-NPs (Selenium nanoparticles)

ROS (Reactive Oxygen Species)

## References

[1] Adibian F, Ghaderi RS, Sabouri Z (2022). Green synthesis of selenium nanoparticles using Rosmarinus officinalis and investigated their antimicrobial activity. BioMetals.

[2] Afshari AR, Jalili-Nik M, Soukhtanloo M (2019). Auraptene-induced cytotoxicity mechanisms in human malignant glioblastoma (U87) cells: role of reactive oxygen species (ROS). EXCLI J.

[3] Akbarnejad-Samani Z, Shamili M, Samari F (2020). The toxicity potential of Ag nanoparticles synthesized from Cordia myxa L. Adv. Hort. Sci..

[4] Alagesan V, Venugopal S (2019). Green synthesis of selenium nanoparticle using leaves extract of withania somnifera and its biological applications and photocatalytic activities. j. Bionanoscience.

[5] Almarzoug MH, Ali D, Alarifi S, Alkahtani S, Alhadheq AM (2020). Platinum nanoparticles induced genotoxicity and apoptotic activity in human normal and cancer hepatic cells via oxidative stress‐mediated Bax/Bcl‐2 and caspase‐3 expression. Environ. Toxicol..

[6] Amiri H, Hashemy SI, Sabouri Z, Javid H, Darroudi M (2021). Green synthesized selenium nanoparticles for ovarian cancer cell apoptosis. Res. Chem. Intermed.

[7] Baharara J, Ramezani T, Divsalar A, Mousavi M, Seyedarabi A (2016). Induction of apoptosis by green synthesized gold nanoparticles through activation of caspase-3 and 9 in human cervical cancer cells. Avicenna J. Med. Biotechnol.

[8] Carneiro BA, El-Deiry WS (2020). Targeting apoptosis in cancer therapy. Nat. Rev. Clin. Oncol.

[9] Cittrarasu V, Kaliannan D, Dharman K (2021). Green synthesis of selenium nanoparticles mediated from Ceropegia bulbosa Roxb extract and its cytotoxicity, antimicrobial, mosquitocidal and photocatalytic activities. Sci. Rep.

[10] Garcia-Oliveira P, Otero P, Pereira AG (2021). Status and challenges of plant-anticancer compounds in cancer treatment. Pharmaceuticals.

[11] Ghaderi RS, Adibian F, Sabouri Z (2021). Green synthesis of selenium nanoparticle by Abelmoschus esculentus extract and assessment of its antibacterial activity. Mater. Technol.

[12] Ghaderi RS, Adibian F, Sabouri Z (2022). Green synthesis of selenium nanoparticle by Abelmoschus esculentus extract and assessment of its antibacterial activity. Mater. Technol.

[13] Hasanin M, Hassan SA, Hashem AH (2021). Green biosynthesis of zinc and selenium oxide nanoparticles using callus extract of Ziziphus spina-christi: characterization, antimicrobial, and antioxidant activity Biomass Convers. Biorefin.

[14] Hashem AH, Salem SS (2022). Green and ecofriendly biosynthesis of selenium nanoparticles using Urtica dioica (stinging nettle) leaf extract: Antimicrobial and anticancer activity. Biotechnol. J.

[15] He L, Zhang L, Peng Y, He Z (2025). Selenium in cancer management: exploring the therapeutic potential. Front. Oncol.

[16] Hosseinpour L, Baharara J, Bostanabad SZ, Darroudi M (2022). Plant-based synthesis of selenium nanoparticles using Cordia myxa fruit extract and evaluation of their cytotoxicity effects. Inorg. Chem. Commun.

[17] Katifelis H, Lyberopoulou A, Mukha I (2018). Ag/Au bimetallic nanoparticles induce apoptosis in human cancer cell lines via P53, CASPASE-3 and BAX/BCL-2 pathways. Artif. Cells, Nanomed Biotechnol.

[18] Khojasteh-Taheri R, Ghasemi A, Meshkat Z, Sabouri Z, Mohtashami M, Darroudi M (2023). Green Synthesis of Silver Nanoparticles Using Salvadora persica and Caccinia macranthera Extracts: Cytotoxicity Analysis and Antimicrobial Activity Against Antibiotic-Resistant Bacteria. Appl. Biochem Biotechnol.

[19] Lin W, Zhang J, Xu J-F, Pi J (2021). The advancing of selenium nanoparticles against infectious diseases. Front. Pharmacol.

[20] Lobb R (2011). Selenium as a Modulator of Efficacy and Toxicity of Chemotherapy and Radiation. Rep. - Univ. Waikato, Antarct. Res. Unit.

[21] Nagaraja K, Hwan OT (2023). Green synthesis of Multifunctional Zinc oxide Nanoparticles from Cordia myxa gum; and their Catalytic Reduction of Nitrophenol, Anticancer and Antimicrobial Activity. Int. J. Biol. Macromol.

[22] Nagaraja K, Prasad B, Almarhoon ZM, Oh TH (2023). Green multifunctional palladium nanoparticles from polysaccharide cordia myxa (CMY) gum: Synthesis, characterization, and antibacterial activity. Colloids Surf.

[23] Nagime PV, Pandey VK, Rajpal C (2025). Biogenic selenium nanoparticles: a comprehensive update on the multifaceted application, stability, biocompatibility, risk, and opportunity. Z Naturforsch C J Biosci.

[25] Pasha A, Kumbhakar DV, Sana SS (2022). Role of biosynthesized Ag-NPs using Aspergillus niger (MK503444 1) in antimicrobial, anti-cancer and anti-angiogenic activities. Front. Pharmacol.

[26] Sabouri Z, Akbari A, Hosseini HA, Khatami M, Darroudi M (2021a). Green-based bio-synthesis of nickel oxide nanoparticles in Arabic gum and examination of their cytotoxicity, photocatalytic and antibacterial effects. Green Chem. Lett. Rev.

[27] Sabouri Z, Rangrazi A, Amiri MS, Khatami M, Darroudi M (2021b). Green synthesis of nickel oxide nanoparticles using Salvia hispanica L (chia) seeds extract and studies of their photocatalytic activity and cytotoxicity effects. Bioprocess Biosyst. Eng.

[28] Sabouri Z, Sabouri S, Moghaddas SSTH, Mostafapour A, Gheibihayat SM, Darroudi M (2022a). Plant-based synthesis of Ag-doped ZnO/MgO nanocomposites using Caccinia macranthera extract and evaluation of their photocatalytic activity, cytotoxicity, and potential application as a novel sensor for detection of Pb2+ ions. Biomass Convers. Biorefin.

[29] Sabouri Z, Sabouri S, Tabrizi Hafez Moghaddas SS, Mostafapour A, Amiri MS, Darroudi M (2022b). Facile green synthesis of Ag-doped ZnO/CaO nanocomposites with Caccinia macranthera seed extract and assessment of their cytotoxicity, antibacterial, and photocatalytic activity. Bioprocess Biosyst. Eng.

[30] Samari F, Parkhari P, Eftekhar E, Mohseni F, Yousefinejad S (2019). Antioxidant, cytotoxic and catalytic degradation efficiency of controllable phyto-synthesised silver nanoparticles with high stability using Cordia myxa extract. J. Exp. Nanosci.

[31] Varlamova EG, Goltyaev MV, Mal’tseva VN (2021). Mechanisms of the cytotoxic effect of selenium nanoparticles in different human cancer cell lines. Int. J. Mol. Sci.

[32] Velayati M, Hassani H, Darroudi M (2021a). Green synthesis of Se-Nanorods using Poly Anionic Cellulose (PAC) and examination of their photocatalytic and cytotoxicity effects. Inorg.

[33] Velayati M, Hassani H, Sabouri Z, Mostafapour A, Darroudi M (2021b). Biosynthesis of Se-Nanorods using Gum Arabic (GA) and investigation of their photocatalytic and cytotoxicity effects. Inorg.

[34] Velayati M, Hassani H, Sabouri Z, Mostafapour A, Darroudi M (2022). Green-based biosynthesis of Se nanorods in chitosan and assessment of their photocatalytic and cytotoxicity effects. Environ.

[35] Wu T, Duan X, Hu C (2019). Synthesis and characterization of gold nanoparticles from Abies spectabilis extract and its anticancer activity on bladder cancer T24 cells. Artif. Cells, Nanomed Biotechnol.

